# Seropositivity to herpes simplex virus type 2, but not type 1 is associated with cervical cancer: NHANES (1999–2014)

**DOI:** 10.1186/s12885-017-3734-2

**Published:** 2017-11-07

**Authors:** Sen Li, Xi Wen

**Affiliations:** 1 School of Life Sciences, Beijing University of Chinese Medicine, Beijing, China; 2 Department of Physiology, LKS Faculty of Medicine, University of Hong Kong, Hong Kong, China; 3 Laboratory of immunogenetics, National Institute of Allergy and Infectious Diseases, National Institutes of Health, Bethesda, MD USA

**Keywords:** Cervical cancer, HSV, HPV, NHANES

## Abstract

**Background:**

Herpes simplex virus types 1 and 2 (HSV1 and HSV2) are infectious agents, and their association with cancer occurrence in human is a controversial topic for decades. We addressed this subject using all available continuous National Health and Nutrition Examination Survey (NHANES) cross-sectional data from 1999 to 2014.

**Methods:**

Eight data cycles (1999–2000, 2001–2002, 2003–2004, 2005–2006, 2007–2008, 2009–2010, 2011–2012, and 2013–2014) were employed, and a sample of 8184 female participants was used in this study according to the availability of cancer history and HSV serostatus.

**Results:**

The seroprevalences of HSV1 and HSV2 were 60.73 ± 0.89 and 25.02 ± 0.64, respectively, and the numbers increased with age (*P* < 0.01). In confounder-adjusted logistic regression analysis, association between HSV1 seropositivity and uterine cancer was identified (adjusted odds ratio-OR_adjusted_ = 6.03; 95% CI: 1.52, 23.87). HSV2 seropositivity was associated with cancer occurrence (OR_adjusted_ = 1.47; 95% CI: 1.01, 2.14), cervical cancer (OR_adjusted_ = 1.72; 95% CI: 1.06, 2.79) and uterine cancer (OR_adjusted_ = 3.49; 95% CI: 1.03, 11.85). Moreover, HSV2 was persistently associated with cervical cancer after further adjusting high-risk human papillomavirus (HPV) as confounder (OR_adjusted_ = 1.90; 95% CI: 1.09, 3.34). Relative risk (RR)-based interaction measurement between HSV2 and HPV on the additive scale suggests higher RR for cervical cancer in participants with seropositivity for HPV only (RR_adjusted_ = 2.98; 95% CI: 1.23, 7.20; *P* = 0.02), HSV2 only (RR_adjusted_ = 2.79; 95% CI: 1.31, 5.96; *P* = 0.01) or both viruses (RR_adjusted_ = 3.44; 95% CI: 1.50, 7.86; *P* < 0.01) when setting participants with seronegativity for both HPV and HSV2 as reference.

**Conclusions:**

The finding of current study provides epidemiological evidence that serostatus of HSV2 can serve as an independent predictor for cervical cancer.

**Electronic supplementary material:**

The online version of this article (10.1186/s12885-017-3734-2) contains supplementary material, which is available to authorized users.

## Background

Cervical cancer represents the fourth most common cancer among women globally: It accounts for 7.9% of total cancer cases in females. 528,000 newly occurred cases and 266,000 deaths were estimated in 2012 [1, 2]. In the United States, the estimated cervical cancer-related emerging cases and deaths are 12,990 and 4120, respectively, in 2016 [3]. Cervical cancer mainly affects countries with low or medium levels of human development, and its incidence rates vary significantly from country to country with a range of 3.8/100,000 in Israel to 48.2/100,000 in Colombia for each year [4]. Early changes in cervix (e.g. squamous intraepithelial lesions) can be detected years before malignancy by screening tests like Pap smear, and such primary screening has resulted in declined incidences of cervical cancer in several countries over the past 30 years. Due to its characteristics of being sexually transmitted, cervical cancer is deemed as sexually transmitted cancer [5]. Indeed, recent changes in sexual behavior have led to increase in risk of HPV infection, thereby elevating cervical cancer incidences in several eastern European countries and former Soviet states [1].

Individuals with HSV1 or HSV2 infection become lifelong carriers. In 2005–2010, the seroprevalences of HSV1 and 2 were 53.9% and 15.7%, respectively, among 14- to 49-year-olds in the United States [6]. As characterized by oral-labial lesions, HSV1 infection is generally transmitted nonsexually while HSV2, as one of the most common sexually transmitted diseases (STDs), is predominantly transmitted from sexual partner with unrecognized or asymptomatic disease. Because of its role in facilitating HIV acquisition and transmission, prevalence and epidemiology of HSV2 have been more frequently described than that of HSV1 [6]. However, it is of note that HSV1 can also be a significant cause of genital herpes in individuals with oral-genital contact [7].

Despite many decades of investigation, the question whether cervical cancer can be induced by herpes viruses is still in debate. HSV2 was hypothesized to be the cervical cancer-inducing sexually-transmitted factor even earlier than HPV [4, 5]: higher rates of HSV2 antibodies have been found in patients with cervical cancer compared to control as early as the second half of twentieth Century [8–10]. However, this opinion has been weakened by some of the epidemiologic studies, along with the lack of detection of HSV2 DNA in cervical tissues [11]. Thus, the proposed aetiological link between HSV2 and cervical carcinoma remains unproved [12]. On the other hand, HSV1 is capable of interfering with DNA repairing and inducing genetic modifications in acute lymphoblastic leukemia [13]. Moreover, HSV1 is found to play a role in thyroid carcinogenesis [14]. To address this subject of an active controversy, current study was performed using the NHANES data with following specific aims: 1) to examine whether HSV1 and 2 were independent risk factors for occurrence of any or female-specific types of cancer, and 2) to evaluate the combined effect of HSV2 and high-risk HPV seropositivity for the development of cervical cancer, and possible interaction between HSV2 and HPV in cervical carcinogenesis.

## Methods

### Study population

Continuous NHANES is a population-based nationwide complex survey to collect and evaluate health and nutrition condition of the non-institutional civilian U.S. population [15]. The survey data is released by U.S. National Centers for Health Statistics (NCHS) biannually for public use since 1999, and NHANES has been approved by National Health Statistics Institutional Review Board. In this study, data from all eight available survey cycles (1999–2014) were employed, which contains a total of 39,755 U.S. female participants. Status of HSV1 and HSV2 infection were measured in the 14–49 years old subgroup in each data-cycle. After excluding subjects with missing (*n* = 27,566) or indeterminate (*n* = 73) HSV1 or HSV2 measurement, a total 12,116 females had HSV serostatus. In this population, subjects who lacked information on whether having cancer (*n* = 1545) or failed to provide specific cancer type (*n* = 7) were also excluded, leaving 10,564 female participants, aged 20 years or older, with cancer status. Next, participants with insufficient information on other variables (*n* = 1949) or received HPV vaccination (*n* = 431) were also removed, leading to a final study population of 8184 females (Additional file 1: Figure S1). For HPV-related analysis, only four data-cycles (2003–2010) were employed due to the availability of serum HPV test, and 4298 female participants were included in the analyses.

### HSV and HPV serostatus

Blood samples were collected from eligible subjects at mobile examination center, and shipped to Emory University or CDC for HSV or HPV analysis, respectively. Serostatus of HSV1 and HSV2 were accessed by solid-phase enzymatic immunodot assay using virus type-specific purified glycoprotein as antigen, and the outcomes were categorized as positive, negative and indeterminate. Competitive Multiplexed Luminex Assay was employed to determine HPV serostatus by simultaneously measuring antibodies against HPV-16 and -18. Readouts of this assay were quantitative, and were presented as arbitrary units (milliMerck units/ml). Threshold for positive results were defined to generate qualitative results (positive-at or above threshold; negative-below threshold) for HPV-16 and -18. Multiplexing the assay has little to no effect on the simplex standard curves for HPV-16 and -18 (the only two types of HPV, as high-risk HPV types, that have been employed in current work), revealing limited cross-reactivity [16]. The detailed methodology is available in the NHANES laboratory procedure manual [17]. For analytic purpose, four additional variables were created: seropositivity for HSV1 or HSV2, both HSV1 and HSV2, HPV (types 16 or 18, as high-risk HPV types), and both HPV and HSV2.

### Cancer status

Cancer status was judged based on answers to the question “Have you ever been told by a doctor or other health professional that you had cancer or a malignancy of any kind?” and its subsequent question “What kind of cancer was it?” in medical condition questionnaire. Occurrence of any type of cancer (any cancer) and four specific female-related cancers were present in this study.

### Other variables

The associations between status of cancer and HSV/HPV seropositivity were adjusted for a series of potential confounding factors: age (20–29, 30–39 or 40–49), race/ethnicity (Non-Hispanic white, Non-Hispanic black or others), education (<high school, high school or >high school), poverty income ratio (PIR; <1, 1 ≤ PIR ≤ median or >median, where medians were computed based on PIR ≥1 for each of the eight data cycles), body mass index (BMI; <25 kgm^−2^ or ≥25 kgm^−2^, where BMI ≥25 kgm^−2^ indicates overweight based on NIH health guidelines).

Current smokers were defined as participants who had smoked ≥100 cigarettes during their lifetimes and reported smoking every day or on some days at the time of interview while former smokers were subjects who smoked ≥100 cigarettes during their lifetimes but were not currently smoking at the time of interview. Participants who smoked less than 100 cigarettes were defined as non-smokers. Current drinkers were participants who had ≥12 drinks of any type of alcoholic beverage in any one year, and had ever drunk over the past 12 months. Former drinkers were subjects who had ≥12 drinks but none in the past year. Subjects who had less than 12 drinks in any one year were defined as non-drinkers. HIV status (positive or negative) was determined by HIV antibody test result. HPV vaccine was first introduced in 2006, and HPV vaccination status was based on the question “Have you ever received one or more doses of the HPV vaccine?” in NHANES 2007–2014 Immunization section. Moreover, weighted percent of HPV vaccination according to survey cycles and age was given in Additional file 2: Figure S2.

### Statistical analysis

The weighted seroprevalences of HSV1 and HSV2 were calculated based on overall data, and data stratified by age, race/ethnicity as well as cancer status. Adjusted odds ratio (OR_adjusted_) and 95% confidence intervals (CIs) for the associations between HSV/HPV and cancer status (any or specific female-related cancer type) were obtained by logistic regression, and the confounders-adjusted models were run with HSV1, HSV2 or HPV16/18 on female-only dataset. Statistical analysis were performed using SAS 9.4 software (SAS Institute Inc., Cary, NC), and the re-calculated sample weights for combined 1999–2014 data, stratification and clustering design variables were integrated into SAS survey procedures.

## Results

Summary statistics of the study population consisting of female participants aged 20–49 years from NHANES 1999–2014 (*n* = 8184) were described in Table 1. The average age of the subjects was 35.44 ± 0.14, and they were equally distributed into three age categories according to the weighted percentage. Only participants aged below 50 years were included in our study due to availability of HSV1 and HSV2 serostatus, leading to relatively young average age. Weighted prevalence of occurrence of any type of cancer or cervical cancer according to age was given in Additional file 3: Figure S3. About 67% of the included participants were non-Hispanic whites while 12% were non-Hispanic black. Other ethnicities represented 21% of study population. Approximately 14% of the study population had been educated less than high school. About 17% of subjects came from low income families (PIR < 1), and 60% of the participants were overweight or obese reflected by BMI ≥ 25 kgm^−2^. Current and former smoker represented 24% and 16% of the study population, respectively, while 66% and 6% were current and former alcohol user, respectively. The incidence of occurrence of any type of cancer (includes, but is not limited to, the following four female-related cancers), breast cancer, cervical cancer, ovarian cancer and uterine cancer were 4.90 ± 0.32, 0.48 ± 0.10, 1.79 ± 0.19, 0.30 ± 0.08 and 0.38 ± 0.09, respectively.

**Table 1:** Weighted characteristics of the study population-NHANES 1999–2014

Variable	Status	Number	Weighted percent	s.e.
Age	20–29 years	2676	28.74	0.71
	30–39 years	2751	33.46	0.71
	40–49 years	2757	37.80	0.73
Race	White	3686	66.73	1.28
	Black	1597	11.91	0.74
	Others	2901	21.36	1.08
Education	<high school	1765	14.47	0.57
	=high school	1698	20.81	0.67
	>high school	4721	64.71	0.94
Poverty income ratio	<1	1944	17.19	0.68
	1 ≤ PIR ≤ median	2789	29.81	0.73
	>median	3451	53.00	1.07
Body mass index	≥25	5362	60.39	0.84
Smoking	Current	1821	24.10	0.70
	Former	1172	16.02	0.64
	Non-	5191	59.88	0.85
Alcohol use	Current	4812	65.56	1.01
	Former	518	5.94	0.33
	Non-	2854	28.49	0.97
HIV	Yes	20	0.16	0.04
Any cancer	Yes	322	4.90	0.32
Breast cancer	Yes	32	0.48	0.10
Cervical cancer	Yes	128	1.79	0.19
Ovarian cancer	Yes	24	0.30	0.08
Uterine cancer	Yes	34	0.38	0.09

Abbreviations: s.e., standard error; HIV, human immunodeficiency virus; NHANES, National Health and Nutrition Examination Survey

**Fig. 1:**
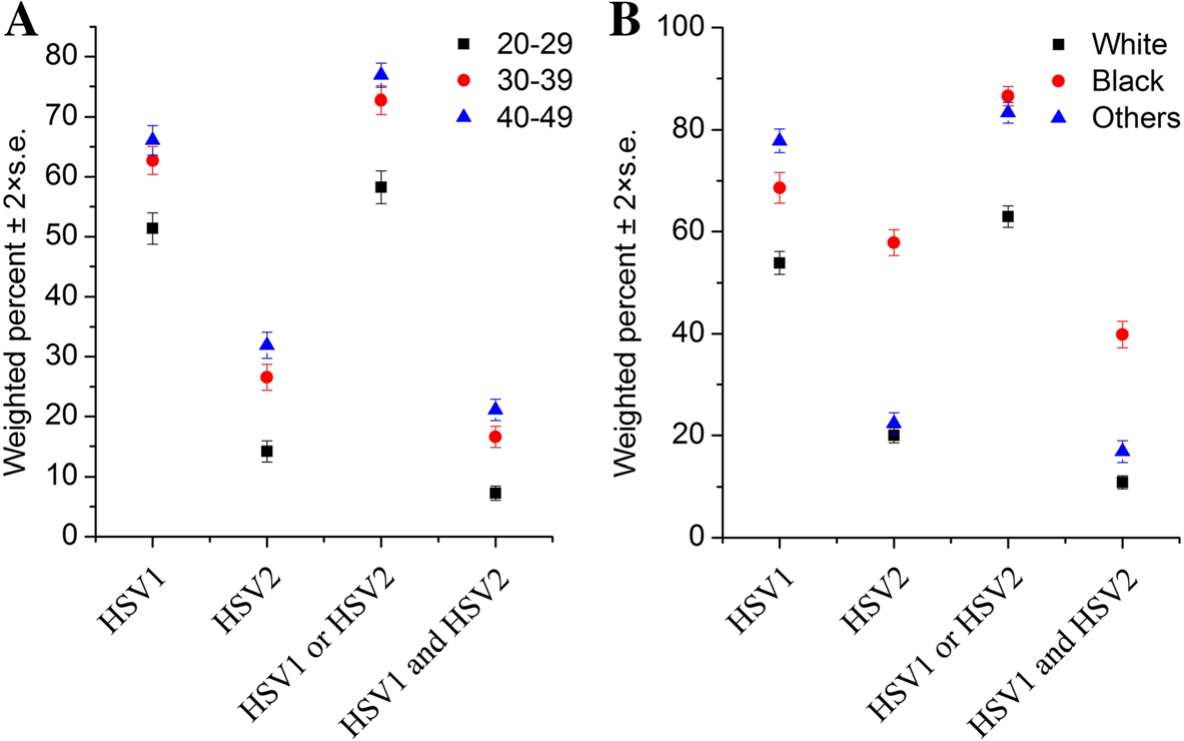
Weighted sero-prevalences of human herpes simplex virus (HSV) type 1 and 2 according to age (**a**) and race (**b**) in study population-NHANES 1999–2014

The seroprevalence of HSV type 1 and 2 were 60.73 ± 0.89 and 25.02 ± 0.64, respectively, for the total population. For the eight data cycles from 1999 to 2014, HSV incidences did not vary a lot with a range of 56.63 ± 2.51 to 64.87 ± 1.35 for HSV1, and 20.93 ± 2.31 to 28.29 ± 2.15 for HSV2 (Additional file 4: Figure S4). Comparing to male, female exhibited significantly higher incidence of having HSV infection for any type (*P* < 0.01) (Additional file 5: Figure S5), which might be partially attributed to more healthcare-seeking behaviour among females. As expected, the prevalence of HSV types increased with age (*P*_trend_ < 0.01 for both types): 66.08 ± 1.24 for HSV1 and 31.90 ± 1.09 for HSV2 among those aged 40–49 years compared to 51.36 ± 1.31 for HSV1 and 14.15 ± 0.88 for HSV2 among those aged 20–29 years (Fig. 1A). Elevated incidence of HSV2 infection was observed among non-Hispanic black compared to non-Hispanic white and other race/ethnicity (*P* < 0.01): 57.83 ± 1.27 vs 20.01 ± 0.74 and 22.35 ± 1.06, respectively (Fig. 1B).

The weighted seroprevalences of both HSV1 and HSV2 were generally higher among sample persons with positive answer for any or specific type of cancer (Table 2), except a slightly lower prevalence of HSV2 among those participants with breast cancer (23.64% vs 25.02%, *P* = 0.88). Crude logistic regression indicated HSV1 seropositivity was associated with cancer occurrence (any cancer) (*P* = 0.04) and uterine cancer (*P* < 0.01). HSV2 seropositivity was associated with cancer occurrence, cervical cancer and uterine cancer, but not breast cancer or ovarian cancer. In confounder-adjusted logistic regression analysis, association between HSV1 seropositivity and uterine cancer was identified (OR_adjusted_ = 6.03; 95% CI: 1.52, 23.87) while cancer occurrence was no longer associated with HSV1 seropositivity (*P* = 0.07). HSV2 seropositivity was associated with cancer occurrence (OR_adjusted_ = 1.47; 95% CI: 1.01, 2.14), cervical cancer (OR_adjusted_ = 1.72; 95% CI: 1.06, 2.79) and uterine cancer (OR_adjusted_ = 3.49; 95% CI: 1.03, 11.85) (Table 3). Adjusting BMI as continuous variable did not alter the results (Additional file 6: Table S1).

**Table 2:** Sero-prevalences of HSV type 1 and 2 according to cancer history-NHANES 1999–2014

Variable	Status	HSV1+ number	HSV1- number	HSV1+ weighted percent	s.e.	*P* value
Any cancer	Yes	229	93	67.17	3.12	0.04
	No	5231	2631	60.39	0.91	
Breast cancer	Yes	24	8	76.16	8.44	0.11
	No	5436	2716	60.65	0.90	
Cervical cancer	Yes	88	40	65.23	5.75	0.44
	No	5372	2684	60.64	0.90	
Ovarian cancer	Yes	21	3	77.82	11.77	0.22
	No	5439	2721	60.68	0.90	
Uterine cancer	Yes	30	4	92.24	4.51	<0.01
	No	5430	2720	60.61	0.90	
Variable	Status	HSV2+ number	HSV2- number	HSV2+ weighted percent	s.e.	*P* value
Any cancer	Yes	119	203	34.26	3.51	<0.01
	No	2144	5718	24.54	0.66	
Breast cancer	Yes	8	24	23.64	9.23	0.88
	No	2255	5897	25.02	0.64	
Cervical cancer	Yes	56	72	40.05	5.41	<0.01
	No	2207	5849	24.74	0.65	
Ovarian cancer	Yes	11	13	44.41	12.95	0.09
	No	2252	5908	24.96	0.65	
Uterine cancer	Yes	15	19	57.99	11.61	<0.01
	No	2248	5902	24.89	0.65	

Abbreviations: HSV1, herpes simplex virus type 1; HSV2, herpes simplex virus type 2; +, positive; −, negative; s.e., standard error; NHANES, National Health and Nutrition Examination Survey

**Table 3:** Associations between HSV types 1 or 2 and cancer status-NHANES 1999–2014

HSV1	aOR	(95% CI)	*P* value
Any cancer	1.32	(0.97–1.79)	0.07
Breast cancer	2.04	(0.78–5.37)	0.14
Cervical cancer	1.04	(0.60–1.80)	0.89
Ovarian cancer	1.57	(0.37–6.64)	0.54
Uterine cancer	6.03	(1.52–23.87)	0.01
HSV2	aOR	(95% CI)	*P* value
Any cancer	1.47	(1.01–2.14)	0.04
Breast cancer	0.69	(0.20–2.40)	0.56
Cervical cancer	1.72	(1.06–2.79)	0.03
Ovarian cancer	1.56	(0.50–4.94)	0.44
Uterine cancer	3.49	(1.03–11.85)	0.04

Abbreviations: HSV1, herpes simplex virus type 1; HSV2, herpes simplex virus type 2; aOR, Adjusted odds ratio; CI, confidence interval; NHANES, National Health and Nutrition Examination Survey

Model was adjusted for age, education, race, poverty income ratio, body mass index, smoking status, alcohol-use status and HIV status

**Fig. 2:**
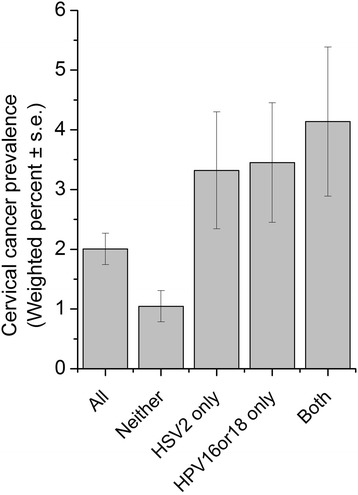
Weighted prevalence of cervical cancers according to HSV2 and high risk-HPV infection status-NHANES 2003–2010

In the following analysis, the interaction between high-risk HPV (types 16 and 18) and HSV types was examined by using data from NHANES 2003–2010 (*n* = 4298) (Table 4). Rao-Scott chi-square test and adjusted logistic models indicated the association between HPV and HSV2 (OR_adjusted_ = 2.49; 95% CI: 1.96, 3.16), but not HSV1. In this subpopulation consisting of four survey cycles, HSV2 was still associated with cervical cancer (OR_adjusted_ = 2.17; 95% CI: 1.28, 3.66) after further adjusting HPV as confounder (OR_adjusted_ = 1.90; 95% CI: 1.09, 3.34). Thus, serostatus of HSV2 could serve as an independent predictor for cervical cancer. In addition, a multiplicative interaction term between high-risk HPV and HSV2 was added to the main model, which indicated no significant result (*P*_interaction_ = 0.14). Relative risk (RR)-based analysis indicated significantly higher RR for cervical cancer in participants with seropositivity for only HPV (RR_adjusted_ = 2.98; 95% CI: 1.23, 7.20; *P* = 0.02), only HSV2 (RR_adjusted_ = 2.79; 95% CI: 1.31, 5.96; *P* = 0.01) or both viruses (RR_adjusted_ = 3.44; 95% CI: 1.50, 7.86; *P* < 0.01) when setting participants with seronegativity for both HPV and HSV2 as reference. However, the further increased RR of seropositivity for both HPV and HSV2 was not statistically significant comparing to those of only HPV (*P* = 0.73) and only HSV2 (*P* = 0.65). In consistent with this analysis, females infected with only high-risk HPV, only HSV2, or both viruses showed significantly higher incidences of cervical cancer (3.45 ± 1.00, 3.32 ± 0.98 and 4.14 ± 1.25, respectively) (Fig. 2).

**Table 4:** Associations between HSV and HPV, and their associations with cervical cancer-NHANES 2003–2010

Dependent variable	Independent variable	aOR	(95% CI)	*P* value
HPV16or18^a^	HSV1	1.03	(0.84–1.27)	0.75
HPV16or18^a^	HSV2	2.49	(1.96–3.16)	<0.01
Cervical cancer^a^	HPV16or18	2.26	(1.14–4.45)	0.02
Cervical cancer^a^	HSV2	2.17	(1.28–3.66)	0.00
Cervical cancer^b^	HSV2	1.90	(1.09–3.34)	0.02

Abbreviations: HSV1, herpes simplex virus type 1; HSV2, herpes simplex virus type 2; HPV16or18, human papillomavirus type 16 or 18; aOR, Adjusted odds ratio; CI, confidence interval; NHANES, National Health and Nutrition Examination Survey

^a^Model was adjusted for age, education, race, poverty income ratio, body mass index, smoking status, alcohol-use status and HIV status

^b^Model was further adjusted for HPV status

## Discussion

The association of cervical cancer with sexual behavior has been illustrated by many investigations since 1842. These studies led to the discovery of cervical cancer-inducing sexually-transmitted agents such as HPV [4]. However, whether HSV is involved in carcinogenesis is still controversial [18]. We examined this long-held idea using NHANES data which represents non-institutional civilian U.S. population (Table 1), and found that prevalence of HSV were usually higher among those female with any or specific type of cancer (Table 2). This is consistent with several clinical findings: higher frequency of occurrence of HSV2 has been observed in invasive cervical cancer cases comparing to cases without neoplastic lesions [19]. Similarly, a significantly higher positivity of HSV2 has been found in women with cervical dysplasia and carcinoma-in-situ [20]. Moreover, compared to HPV DNA-positive normal controls, seropositivity of HSV2 is significantly greater in cases with cervical cancer [21]. As reported by an epidemiological study, higher levels of HSV2 antibodies have been found more common in cervical cancer patients [22]. Our subsequent multivariate logistic regression analysis indicated that 1) HSV1 seropositivity was associated with uterine cancer, and 2) HSV2 seropositivity was associated with cancer occurrence, cervical cancer and uterine cancer (Table 3). Indeed, high HSV2 prevalence has been related to high-risk area of cervical cancer [23]. For instance, The highest incidence of cervical cancer was in Africa as reported by GLOBOCAN project which provides contemporary estimates of the incidence of major types of cancer [24]. Such high incidence of cervical cancer may be partially attributed to high prevalence of HSV2 (around 70%) among women in Africa [25]. Moreover, an increased risk of cervical cancer in women with antibodies against HSV2 has been suggested by several studies [26–28]. One study showed that those female with positive HSV2 testing results have a 60% increased risk of cervical cancer in contrast to HSV2 seronegative women [27], while another study indicated this risk can be two- to three-fold higher [29]. In a prospective study, incidences of cancer are directly related to the prevalence of genital herpes symptoms [30]. Furthermore, immuno-cytochemistry suggested HSV2 is probably associated with squamous cell carcinoma cervix and carcinoma in situ [31], indicating that HSV2 may play separate etiologic roles in cervical malignant transformation [32]. Globally, the possible association between HSV2 seroprevalence and cervical cancer has been known to differ markedly across various countries [21]. A study in Nepal indicates that HSV2 is detected in 11% cases of cervical intraepithelial neoplasia (CIN) I, 33% cases of CIN III and 40% cases of carcinoma cervix with a gradual increasing antibody titre [33]. In India, HSV, diagnosed in cytology smears, is largely associated with squamous intraepithelial lesion and carcinoma cervix even with higher affinity compared to HPV [34]. The independent effect of HSV2 seropositivity in cervical malignant transformation is also found in Latin American [27]. In contrast, such association is not identified in the south of Brazil [35], China [36], Jamaica [37] and Nordic countries [38]. These inconsistencies may also derive from differences in detection methods and severity of cervical lesions [35]. It is of note that HSV genes may be necessary for the initiation, but not progression in cervical malignant transformation as proposed by “*hit and run*” mechanism [39].

The role of HSV2 in in vivo carcinogenesis has been confirmed in animal models. Prolonged exposure of formalin or ultraviolet inactivated HSV1 or 2 leads to premalignant or malignant lesions in mouse cervix [40]. HSV2 DNA transfection also induces cervical neoplasia reflected by premalignant or malignant lesions on vaginal smears with similar cytologic and histologic characteristics of that occurred in women [41]. At cellular and molecular level, transfection with HSV2 morphological transforming region III (MTR III) leads to morphological transformation in HPV-immortalized keratinocytes, and lesions can be developed when injecting these cells to nude mice [42]. Moreover, HSV2-infection in cervical cancer cell line rapidly increases the proportions of DNA-synthesizing G1- and S-phase cells, resulting in unscheduled DNA synthesis [43]. Accumulative evidences from biological experiments suggest that HSV gene products interfere with cell cycle control, induce accumulation of genetic abnormalities and destabilize host genome, indicating a role of HSV in cellular malignant transformation.

On the other hand, the causative role of HPV in cervical carcinogenesis appears to be incontrovertible [44, 45]. 40 of 120 types of HPVs discovered to date are involved in anogenital infections [46], in which HPV types 16 and 18 are the major etiological factors of cervical cancer causing 70% of all cases [47]. The mechanism of HPV-mediated carcinogenesis is relatively clear. HPV integration into the host DNA disrupts E2 gene and results in the removal of its transcriptional repression on E6/E7, which is known to immortalize primary cervical cells and human foreskin keratinocytes in culture [48, 49]. More specifically, the E6 oncoprotein of high-risk HPVs effectively binds to p53 and leads to its degradation via ubiquitin mediated pathway. This disabled chromosomal repair in damaged cells. Continuous division of damaged cells increases chromosomal mutations, and hence the development of carcinoma [18]. However, HPV infection alone is neither sufficient nor necessary for malignant transformation [50]. In females with high-risk HPV infection, only a small proportion will develop invasive cervical cancer over years [35]. It is consistent with evidence from cell experiments that HPV genes can immortalize normal cells, but are incapable of their transformation [50]. Numerous evidences of cervical cancer occurrences in HPV-negative population also deny the postulate that about 90% of cervical cancer is caused by HPV [4, 51]. Thus, additional factors besides HPV may be necessary to convert dysplastic lesions to carcinomas, reflecting the multifactorial etiology of cervical carcinogenesis [52].

Next, we want to investigate whether both HSV2 and HPV infection have synergistic functions in cancer development. STDs normally occur with high level of concordance, which is supported by our result showing that HPV was associated with HSV2, but not HSV1 (Table 4). Furthermore, relative risk-based interaction measurement between HSV and HPV on the additive scale suggests that participants with seropositivity for both HPV and HSV2 showed a relatively higher risk for cervical cancer (Table 4). Indeed, synergistic interactions between HPV and HSV have been reported in case-control study [27]. HSV may interact directly with HPV to facilitate its integration and amplification in host cells, thereby elevating the risk of cervical cancer [53–55]. Mechanistically, co-infection HSV2 with HPV interfere local immune responses, which increases the likelihood of HPV-associated lesions progression [37]. More importantly, tumorigenic growth can be induced by HSV in cells immortalized by HPV16 [42, 52], and HSV2 transfection is also required for tumours induction in mice by HPV16/18 transformed cells [56]. Thus, HSV2 play roles in immortalisation and cooperates in the process of malignant transformation.

In order to exclude the possibility that infection with HSV2 serves as surrogate marker of HPV exposure, and is of no separate aetiological significance [26, 57], we further adjusted HPV as confounder. The results consistently indicated that HSV2 seropositivity was associated with cervical cancer (Table 4). The role of HSV2 as independent predictor for cervical cancer is supported by several studies. Zhao et al. reveals that HSV2, but not HSV1, infection or co-infection with HPV is involved in cervical malignant transformation [58]. The association between HSV seropositivity and CIN grade II-III exists, and cannot be explained by HPV [59]. Moreover, HSV2 antibodies were associated with cervical cancer in HPV-negative cases, revealing HSV2 as primary viral agent [60].

## Conclusions

The observed associations between HSV seropositivity and cancer occurrence in this study merits further investigation. Unlike HPV, there is currently no effective vaccine against herpes virus, which makes current study particularly important. More fundamental research on cervical carcinogenesis is needed to elucidate the etiology of such multifactorial disease and better prevent, treat and cure cervical cancer that causes high morbidity and mortality among females globally.

## Additional files

Additional file 1: Figure S1. NHANES participant enrollment flowchart including exclusion criteria. (JPEG 814 kb)

Additional file 2: Figure S2. Weighted percent of human papillomavirus (HPV) vaccination according to NHANES survey cycles (A) and age (B) in female population-NHANES 2007–2014 (*n* = 4375). (JPEG 203 kb)

Additional file 3: Figure S3. Weighted prevalences of occurrence of any type of cancer (A) and cervical cancer (B) according to age in study population-NHANES 1999–2014. (JPEG 250 kb)

Additional file 4: Figure S4. Weighted sero-prevalences of human herpes simplex virus (HSV) type 1 and 2 according to NHANES survey cycles. (JPEG 286 kb)

Additional file 5: Figure S5. Weighted sero-prevalences of human herpes simplex virus (HSV) type 1 and 2 according to gender in population consisting of both genders (*n* = 16,734)-NHANES 1999–2014. (JPEG 211 kb)

Additional file 6: Table S1. Associations between HSV types 1 or 2 and cancer status-NHANES 1999–2014. (DOCX 14 kb)

## Abbreviations

BMI: body mass index
CDC: Centers for Disease Control and Prevention
CI: confidence interval
CIN: Cervical intraepithelial neoplasia
CIs: confidence intervals
HIV: human immunodeficiency virus
HPV: human papillomavirus
HPV16or18: human papillomavirus type 16 or 18.
HSV: herpes simplex virus
MTR III: morphological transforming region III
NCHS: National Centers for Health Statistics
NHANES: National Health and Nutrition Examination Survey
NIH: National Institutes of Health
OR_adjusted_: adjusted odds ratio
PIR: poverty income ratio
RR: Relative risk
s.e.: standard error
STDs: sexually transmitted diseases
