# Supplementary figures and images for: Seropositivity to herpes simplex virus type 2, but not type 1 is associated with cervical cancer: NHANES (1999–2014)
Source: BMC Cancer. 2017 Nov 7;17:726. doi: 10.1186/s12885-017-3734-2 (PMC5678804; doi:10.1186/s12885-017-3734-2)

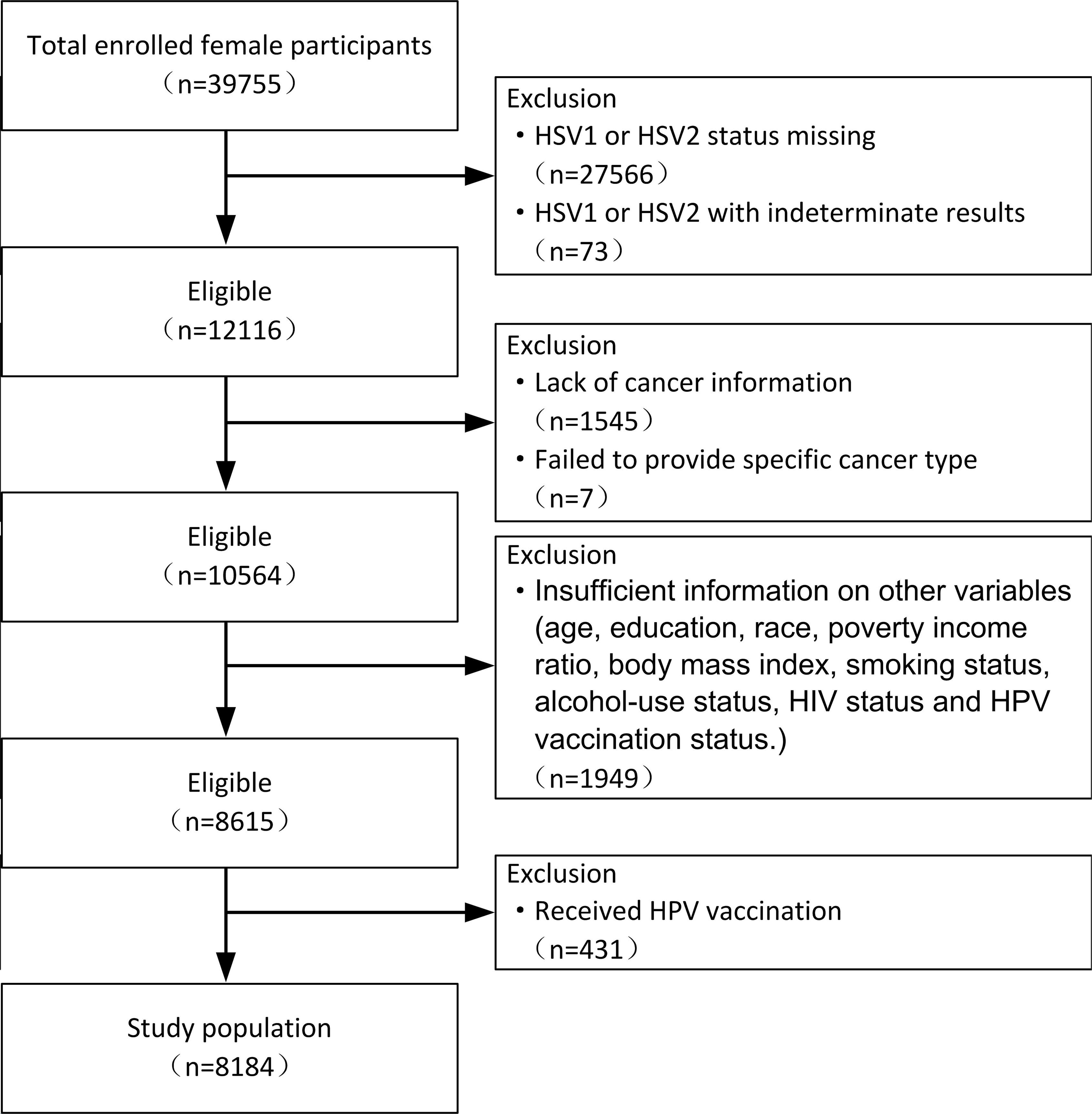

Supplement: Supplementary file 1 — NHANES participant enrollment flowchart including exclusion criteria. (JPEG 814 kb) [file 12885_2017_3734_MOESM1_ESM.jpg]

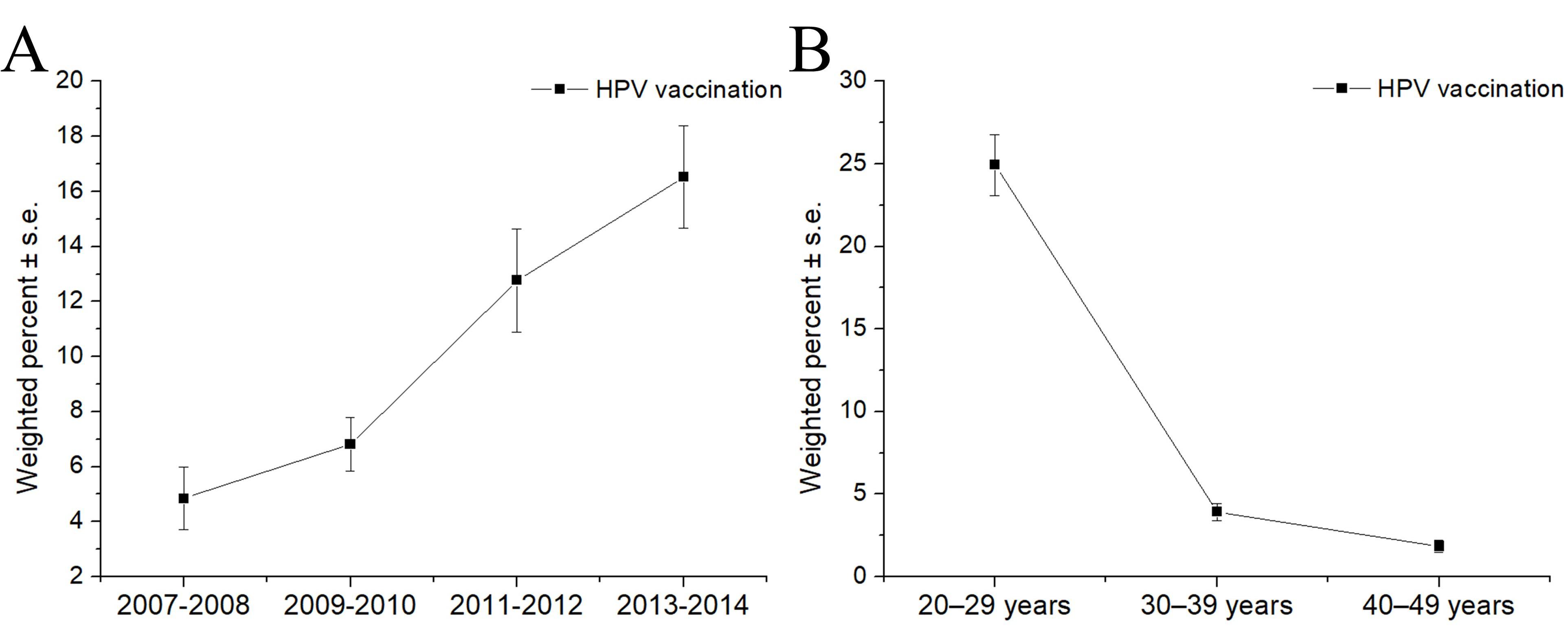

Supplement: Supplementary file 2 — Weighted percent of human papillomavirus (HPV) vaccination according to NHANES survey cycles (A) and age (B) in female population-NHANES 2007–2014 (n = 4375). (JPEG 203 kb) [file 12885_2017_3734_MOESM2_ESM.jpg]

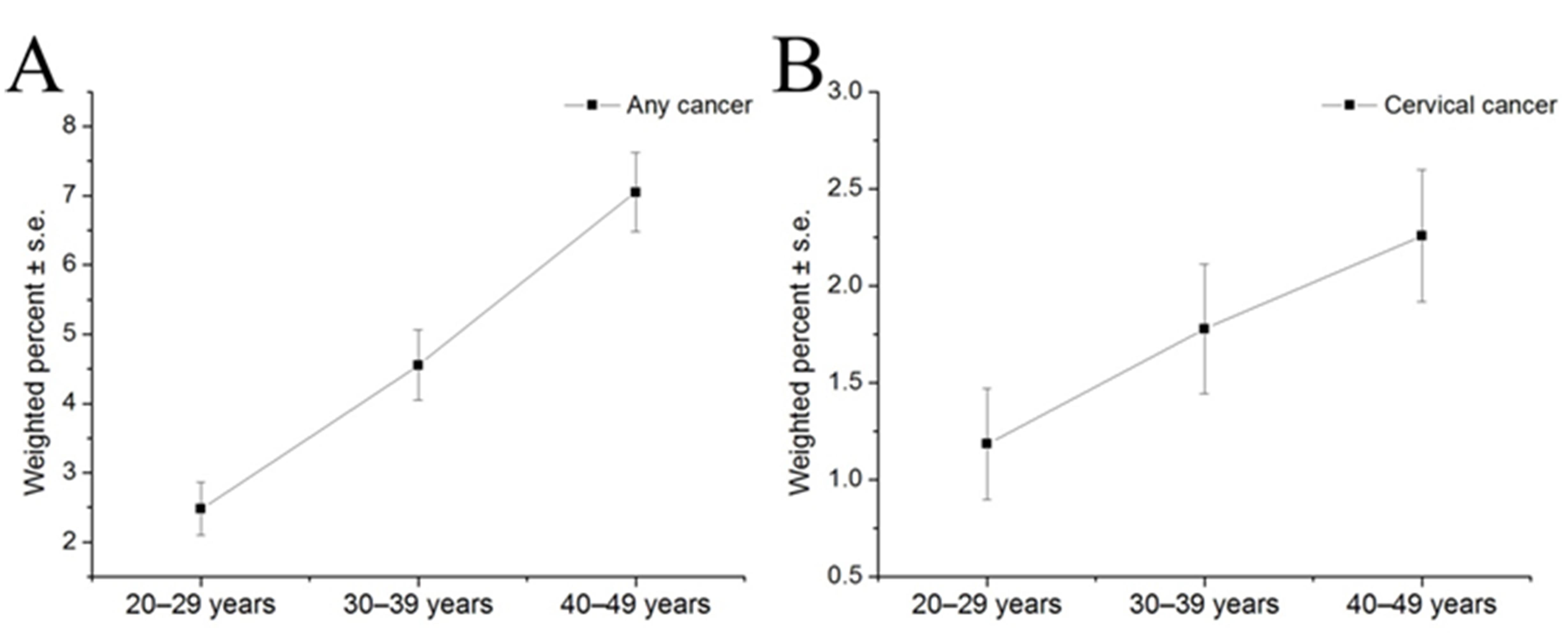

Supplement: Supplementary file 3 — Weighted prevalences of occurrence of any type of cancer (A) and cervical cancer (B) according to age in study population-NHANES 1999–2014. (JPEG 250 kb) [file 12885_2017_3734_MOESM3_ESM.jpg]

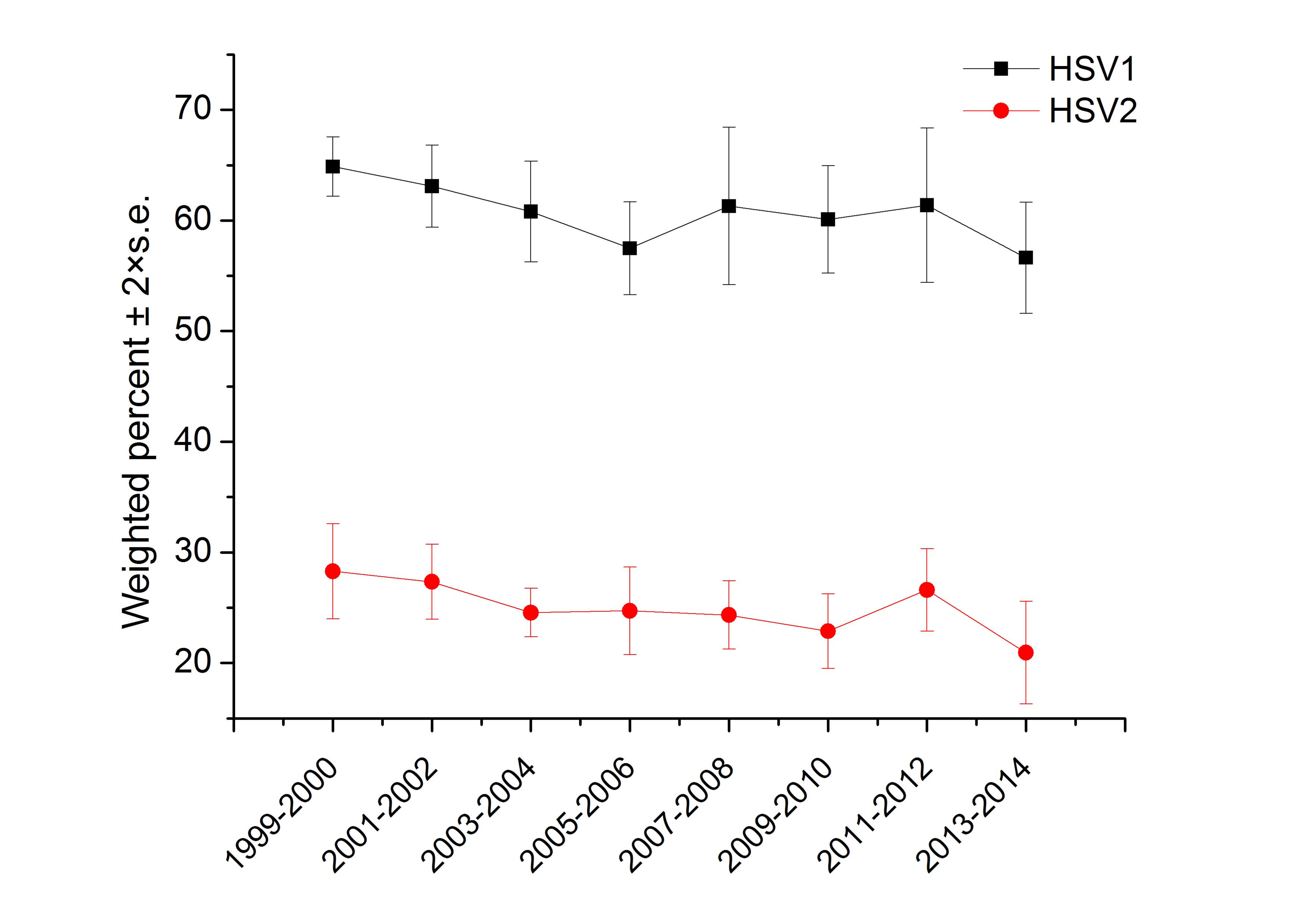

Supplement: Supplementary file 4 — Weighted sero-prevalences of human herpes simplex virus (HSV) type 1 and 2 according to NHANES survey cycles. (JPEG 286 kb) [file 12885_2017_3734_MOESM4_ESM.jpg]

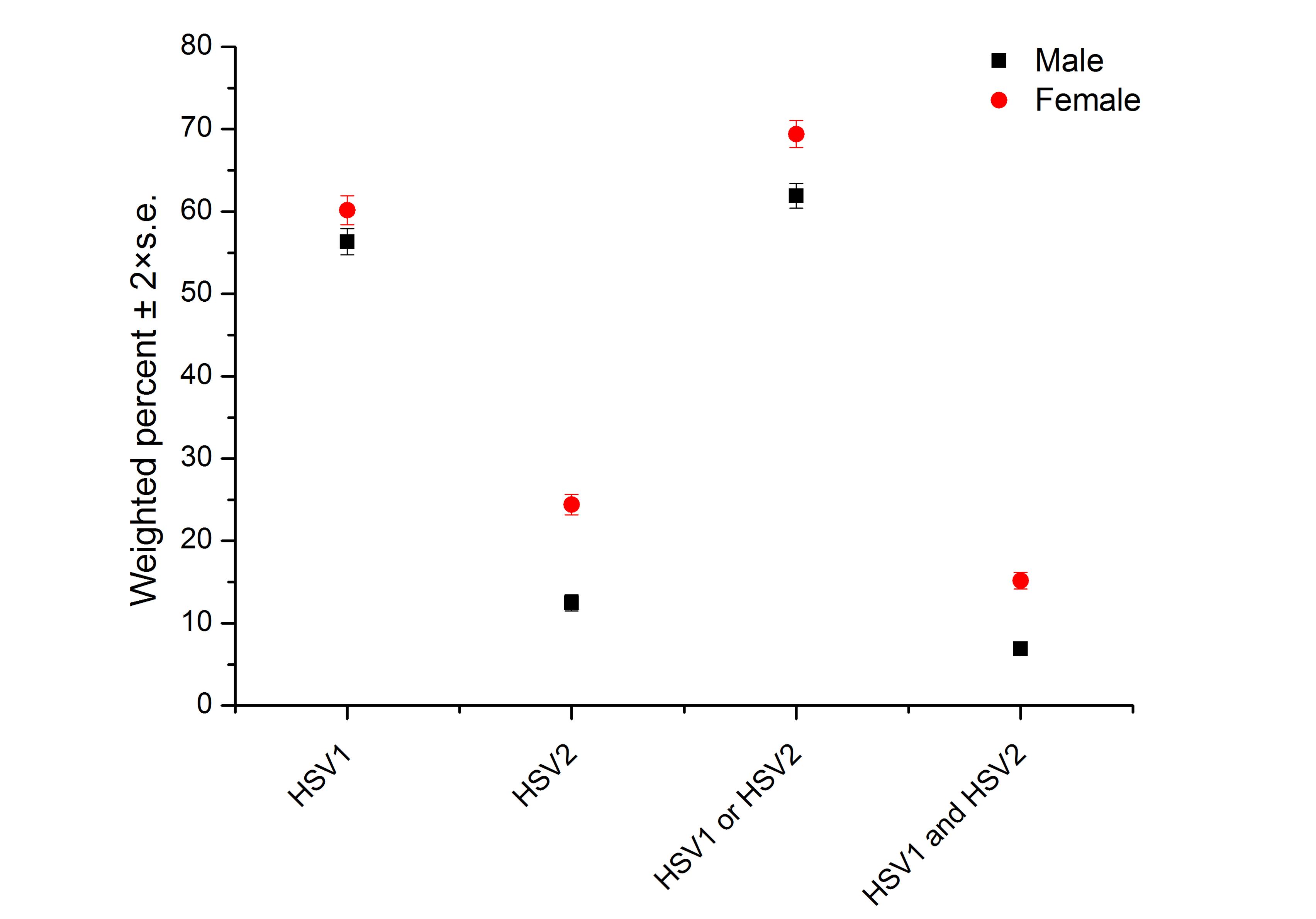

Supplement: Supplementary file 5 — Weighted sero-prevalences of human herpes simplex virus (HSV) type 1 and 2 according to gender in population consisting of both genders (n = 16,734)-NHANES 1999–2014. (JPEG 211 kb) [file 12885_2017_3734_MOESM5_ESM.jpg]
