# Supplementary material for: Seropositivity to herpes simplex virus type 2, but not type 1 is associated with cervical cancer: NHANES (1999–2014)
Source: BMC Cancer. 2017 Nov 7;17:726. doi: 10.1186/s12885-017-3734-2 (PMC5678804; doi:10.1186/s12885-017-3734-2)
Supplement: Supplementary file 6 — Associations between HSV types 1 or 2 and cancer status-NHANES 1999–2014. (DOCX 14 kb) [file 12885_2017_3734_MOESM6_ESM.docx]

Table S1. Associations between HSV types 1 or 2 and cancer status-NHANES 1999-2014.

| HSV1 | aOR | (95% CI) | *P* value |
| --- | --- | --- | --- |
| Any cancer | 1.32 | (0.97-1.78) | 0.07 |
| Breast cancer | 2.01 | (0.77-5.29) | 0.15 |
| Cervical cancer | 1.04 | (0.60-1.81) | 0.89 |
| Ovarian cancer | 1.59 | (0.38-6.72) | 0.53 |
| Uterine cancer | 5.95 | (1.52-23.37) | 0.01 |
| HSV2 | aOR | (95% CI) | *P* value |
| Any cancer | 1.47 | (1.01-2.14) | 0.04 |
| Breast cancer | 0.68 | (0.20-2.33) | 0.53 |
| Cervical cancer | 1.72 | (1.06-2.79) | 0.03 |
| Ovarian cancer | 1.56 | (0.50-4.90) | 0.44 |
| Uterine cancer | 3.47 | (1.02-11.79) | 0.04 |
| Abbreviations: HSV1, herpes simplex virus type 1; HSV2, herpes simplex virus type 2; aOR, Adjusted odds ratio; CI, confidence interval; NHANES, National Health and Nutrition Examination Survey.  Model was adjusted for age, education, race, poverty income ratio, body mass index (continuous variable), smoking status, alcohol-use status and HIV status. | | | |
